# Altered Spontaneous Glutamatergic and GABAergic Activity in the Peritumoral Cortex of Low-Grade Gliomas Presenting With History of Seizures

**DOI:** 10.3389/fnins.2021.689769

**Published:** 2021-06-28

**Authors:** Soumil Dey, Ramesh Sharanappa Doddamani, Aparna Banerjee Dixit, Manjari Tripathi, Meher Chand Sharma, P. Sarat Chandra, Jyotirmoy Banerjee

**Affiliations:** ^1^Department of Neurosurgery, All India Institute of Medical Sciences, New Delhi, India; ^2^Dr. B. R. Ambedkar Centre for Biomedical Research, University of Delhi, New Delhi, India; ^3^Department of Neurology, All India Institute of Medical Sciences, New Delhi, India; ^4^Department of Pathology, All India Institute of Medical Sciences, New Delhi, India; ^5^Department of Biophysics, All India Institute of Medical Sciences, New Delhi, India

**Keywords:** low-grade glioma, seizure, peritumoral tissue, glutamatergic activity, GABAergic activity

## Abstract

The peritumoral regions of WHO grade II gliomas, like astrocytoma and oligodendroglioma, have been reported to show epileptiform activities. An imbalance of glutamatergic and GABAergic mechanisms is primarily responsible for the generation of epileptiform activities. Here we have compared the electrophysiological properties of pyramidal neurons in intraoperative peritumoral specimens obtained from glioma patients with (GS) and without (GN) a history of seizures at presentation. Histology and immunohistochemistry were performed to assess the infiltration of proliferating cells at the peritumoral tissues. Whole-cell patch clamp technique was performed to measure the spontaneous glutamatergic and GABAergic activity onto pyramidal neurons in the peritumoral samples of GS (*n* = 11) and GN (*n* = 15) patients. The cytoarchitecture of the peritumoral tissues was devoid of Ki67 immuno-positive cells. We observed a higher frequency of spontaneous glutamatergic and GABAergic activities onto pyramidal neurons of the peritumoral samples of GS patients. Our findings suggest that, in spite of similar histopathological features, the pyramidal neurons in the peritumoral samples of GS and GN patients showed differences in spontaneous excitatory and inhibitory synaptic neurotransmission. An alteration in postsynaptic currents may contribute to the spontaneous epileptiform activity in GS patients.

## Introduction

Low-grade gliomas (LGG), like oligodendrogliomas (ODG) and astrocytomas, usually present with a history of multiple seizures apart from other neurological symptoms (van Breemen et al., 2007). The seizures primarily originate from the neocortex surrounding gliomas (Haglund et al., 1992; Berger et al., 1993). One pertinent hypothesis of seizure generation is an imbalance between excitatory and inhibitory synaptic transmissions mediated by glutamate (NMDA, AMPA/kainate) and GABA receptors. The downregulation of excitatory amino acid transporters (Ye et al., 1999; Takano et al., 2001) and the upregulation of cystine–glutamate transporter in glioma cells result in a higher peritumoral glutamate concentration than in the physiological scenario (Savaskan et al., 2008; Marcus et al., 2010). The mutation of isocitrate dehydrogenase 1 (IDH-1) gene leads to the accumulation of D-2-hydroxyglutarate in glioma cells, which acts as agonist of glutamate receptors when the extracellular concentration of glutamate is not increased (Dang et al., 2009; Chen et al., 2017), contributing to hyperexcitability and seizure generation (Huberfeld and Vecht, 2016). Intracellular chloride homeostasis is also disturbed in glioma cells due to the upregulation of Na–K–2Cl cotransporter 1 (NKCC1) and the downregulation of K–Cl cotransporter 2 (KCC2) (Pallud et al., 2014). Dysregulated chloride homeostasis contributes to abnormal GABAergic signaling and depolarizes pyramidal neurons in peritumoral tissues, thereby contributing to seizure generation (Huberfeld and Vecht, 2016). We have previously shown that the spontaneous glutamatergic activity in cortical tissue samples obtained from mesial temporal lobe epilepsy patients was higher compared to that in the case of peritumoral tissue samples obtained from glioma patients without a history of seizures (Banerjee et al., 2015, 2017, 2020). The aim of this study was to compare the electrophysiological properties of pyramidal neurons in the peritumoral cortex of astrocytoma and ODG patients with a history of seizures (GS) with that in the case of patients without seizures (GN). Here we hypothesized that, despite similar histopathological features, there would be differences in spontaneous glutamatergic and GABAergic neurotransmission when comparing pyramidal neurons from the peritumoral samples of GS patients with that in the case of GN patients.

## Materials and Methods

This study was carried out in accordance with the recommendations of the institutional ethics committee (ref. no. OP-12/09.05.2011 and ref. nos. IEC/553/03.11.2017, AA-2/29.12.2017, AA-6/02.02.2018, and RP-4/2018), AIIMS. A written and informed consent was obtained for all the patients. A total of 77 patients whose radiological features were suggestive of LGG were operated between 2015 and 2019 at our institution. The intraoperative peritumoral cortical tissues obtained from all the patients (as part of the planned surgical resection) were collected, labeled, and processed. The peritumoral area was defined as the area immediately beside the tumor. Brain samples were part of the planned surgical resection, and no additional brain tissue was removed for the experiments reported in this study. After resection, the brain tissues were collected in well-carbogenated (95% O_2_ + 5% CO_2_) artificial cerebro-spinal fluid (ACSF). We included only the low-grade gliomas (WHO grade II) based on the final histopathological diagnosis as well as IDH-1 status for the purpose of this study. The patients were further categorized into LGGs with seizure [GS (*n* = 11)] and without seizures [GN (*n* = 15)] (Table 1).

**TABLE 1:** Clinical details of low-grade glioma patients with seizures.

Patient ID	Age (years)	Sex	Pathology	AEDs	Seizure semiology/symptoms	Tumor location	Mutated IDH1 (IDH1 R132H) immunostaining
GS1	37	M	Oligodendroglioma, (WHO grade II)	PHE	Generalized tonic–clonic seizures^a^	Right temporal	Positive
GS2	15	F	Astrocytoma, (WHO grade II)	LEV	Generalized tonic–clonic seizures^a^	Left fronto-parietal	Positive
GS3	29	M	Oligodendroglioma, (WHO grade II)	CLO, LEV	Recurrent focal seizures with secondary generalization^b^	Left fronto-parietal	Positive
GS4	19	M	Oligodendroglioma, (WHO grade II)	LEV	Focal onset seizures^a^	Right fronto-temporal	Positive
GS5	35	F	Astrocytoma, (WHO grade II)	LEV	Generalized tonic–clonic seizures^a^	Left frontal	Positive
GS6	32	F	Astrocytoma, (WHO grade II)	OXC	Right focal seizures^a^	Left fronto-temporal	Positive
GS7	53	M	Oligodendroglioma, (WHO grade II)	PHE, VAL	Multiple focal onset seizures involving the left upper limb^b^	Right fronto-parietal	Positive
GS8	53	M	Oligodendroglioma, (WHO grade II)	CAR	Aura of fear with speech arrest and focal onset seizures with loss of awareness^a^	Left temporal	Positive
GS9	47	M	Oligodendroglioma, (WHO grade II)	LEV	Focal seizures with secondary generalization^a^	Left parietal	Positive
GS10	45	F	Astrocytoma, (WHO grade II)	VAL	Generalized tonic–clonic seizures^a^	Right temporal	Positive
GS11	31	M	Oligodendroglioma, (WHO grade II)	PHE	Multiple episodes of focal seizures^a^	Right insular	Positive
GN1	39	F	Astrocytoma (WHO grade II)	N.A.	Recurrent vomiting and altered sensorium	Right parietal	Negative
GN2	25	M	Oligodendroglioma (WHO grade II)	N.A.	Headache, tinnitus, visual deterioration, and diplopia	Left parieto-occipital	Negative
GN3	39	M	Astrocytoma (WHO grade II)	N.A.	Headache and right hemiparesis	Right temporal	Negative
GN4	19	M	Astrocytoma (WHO grade II)	N.A.	Decreased sensation and progressive weakness involving the right hand with deviation of the face, inability to speak, and occasional headache	Left posterior frontal	Negative
GN5	35	M	Astrocytoma (WHO grade II)	N.A.	Headache and recurrent vomiting, right hemiparesis	Left Insular	Negative
GN6	3	F	Oligodendroglioma (WHO grade II)	N.A.	Episodic headache with nausea and vomiting	Right Frontal	Negative
GN7	50	M	Astrocytoma (WHO grade II)	N.A.	Recurrent frontal headache	Left frontal	Negative
GN8	37	M	Astrocytoma (WHO grade II)	N.A.	Headache, decreased vision, and difficulty in walking	Left frontal	Negative
GN9	45	F	Astrocytoma (WHO grade II)	N.A.	Headache, nausea, vomiting, and right hemiparesis	Left fronto-parietal	Negative
GN10	25	M	Astrocytoma (WHO grade II)	N.A.	Parietal headache	Left parieto-occipital	Negative
GN11	36	M	Astrocytoma (WHO grade II)	N.A.	Headache and multiple vomiting	Left frontal	Negative
GN12	65	M	Astrocytoma (WHO grade II)	N.A.	Features of raised intracranial pressure	Right frontal	Negative
GN13	28	F	Astrocytoma (WHO grade II)	N.A.	Recurrent headache	Left fronto-parietal	Negative
GN14	39	M	Oligodendroglioma (WHO grade II)	N.A.	Headache, vomiting, and behavioral changes	Left frontal	Negative
GN15	35	F	Astrocytoma (WHO grade II)	N.A.	Headache with nausea	Right fronto-temporal	Negative

*CLO, clobazam; VAL, valproate; LEV, levetiracetam; OXC, oxcarbazepine; CAR, carbamazepine; PHE, phenytoin. ^*a*^Well controlled with medication. ^*b*^Partial control with medication.*

### Histology and Immunohistochemistry

After fixation, the tissues were dehydrated in an increasing gradient of ethanol and xylene (70, 90, and 100%, xylene; 1 h in each) and embedded into paraffin blocks. The paraffin-embedded tissues were then cut into 6-μM-thick sections with a microtome and collected on poly L-lysine-coated slides (Sigma). The sections were deparaffinized with xylene and rehydrated with a decreasing gradient of ethanol (100, 90, and 70%; 1 h each). Subsequently, the sections were used for hematoxylin–eosin staining for cytoarchitecture. For immunohistochemistry, the sections were subjected to antigen retrieval with sodium citrate (pH 6.0, 30 min, 90°C), followed by masking endogenous peroxidase activities with 1% methanolic H_2_O_2_ (30 min). Primary antibody incubation was against Ki67 (rabbit monoclonal, 1:100, Abcam ab16667) and Olig2 (rabbit monoclonal, 1:200, Abcam ab109186), overnight at 4°C in a humid chamber, followed by incubation with biotinylated secondary antibody (1:1,000; Vector Laboratories ba-1000) for 60 min at room temperature. The signals were amplified using an avidin-biotinylated enzyme complex kit (Vector), and colors were generated using 3,3′-diaminobenzidine chromogen (Sigma) (Khanna et al., 2018).

### Patch Clamp Electrophysiology

Within 15 mins of resection, the peritumoral samples obtained from the GN and GS patients were brought to the laboratory in well-carbogenated, ice-cold ACSF composed of NaCl–125 mM, KCl–2.5 mM, CaCl_2_–2 mM, NaHCO_3_–25 mM, NaH_2_PO_4_–1.25 mM, MgCl_2_–1 mM, and glucose–25 mM (Alkondon et al., 2000). The size of the samples was generally within the range of 1 cm^3^. Slices (350-μm thickness) were prepared using a vibrating blade microtome. The slices were prepared by making tangential cuts to the outer surface of the cortical specimens and were stored at room temperature in carbogenated ACSF. Pyramidal neurons from layer III or IV were used for this study. The slices were transferred to the recording chamber and perfused with carbogenated ACSF at a rate of 2 ml/min. Infrared-assisted video-microscopy with differential interference contrast was used to morphologically identify pyramidal neurons located in the slice preparations. Normal-appearing pyramidal neurons were morphologically identified by its pyramid-like and a single, thick, tapering apical dendrite. The cells on the surface slice preparations were usually dead, so we used the pale-looking pyramidal neurons from layer III or IV for our studies. Patch pipettes (borosilicate capillary tubes; OD, 1.2 mm; resistance, 3–7 MΩ) were filled with an internal pipette solution (HEPES, 10 mM; MgCl_2_, 2 mM; Cs-methanesulfonate, 130 mM; EGTA, 10 mM; CsCl, 10 mM) (Alkondon et al., 2000). Whole-cell patch clamp recordings were performed from the pyramidal neurons in voltage clamp mode using an amplifier (Axopatch200B). Passive membrane properties and postsynaptic currents (PSCs) were recorded in pCLAMP 10 software (Molecular Devices, United States). Spontaneous excitatory and inhibitory PSCs (sEPSCs/sIPSCs) were recorded at -70 and 0 mV holding potentials, respectively, from the identified pyramidal neurons. The access resistance was generally from 10 and 20 MΩ for all recordings. All the events were recorded at 20–22°C.

After recording, 5-min epochs of spontaneous PSCs (sEPSCs/sIPSCs) were analyzed in the Clampfit module of pCLAMP software. Frequency, peak amplitude, rise time (10–90%), and the decay time constant (*τ*_*d*_) of the sEPSCs and sIPSCs were measured. The threshold amplitude for detecting sEPSCs was set at -5 pA and for sIPSCs at +10 pA. The recorded PSCs were visually rechecked to reduce inaccuracies. We have manually included PSCs which demonstrated a normal synaptic waveform. Events which show a steeply rising and exponential decay phase were included for kinetic analysis. Double- and multiple-peak events were excluded for the calculation of kinetic properties (rise time and decay time constant) but included for the calculation of frequencies.

Statistical analysis was performed in Sigmaplot 13.0 (Systat Software). Evaluation between two groups was performed by Mann–Whitney test. Kolmogorov–Smirnov test was used to check the cumulative distribution of events. Data are shown as mean ± SEM. *p* < 0.05 was considered significant.

## Results

### Patients

A total of 26 patients with a final diagnosis of LGG (WHO grade II) were included in this study, comprising of 17 male and nine female patients. The GS group included seven male and four female patients, and the GN group included 10 male and five female patients. The mean age of patients in the GS group was 36.00 ± 3.82 years and for the GN group was 34.67 ± 3.82 years (*p* = 0.881, Mann–Whitney test). The histopathology was ODG in seven and astrocytoma in four patients in the GS group, while in the GN group ODG constituted three and astrocytoma accounted for 12 patients (Table 1). All the GS patients demonstrated a positive status for mutated IDH-1. Among the 11 patients in the GS group, nine had well-controlled seizures with a single antiepileptic drug (AED). Two patients continued to have seizures (although with reduced frequency) even with two AEDs. We have not observed any correlation between the type of glioma and seizure frequency.

**FIGURE 1:**
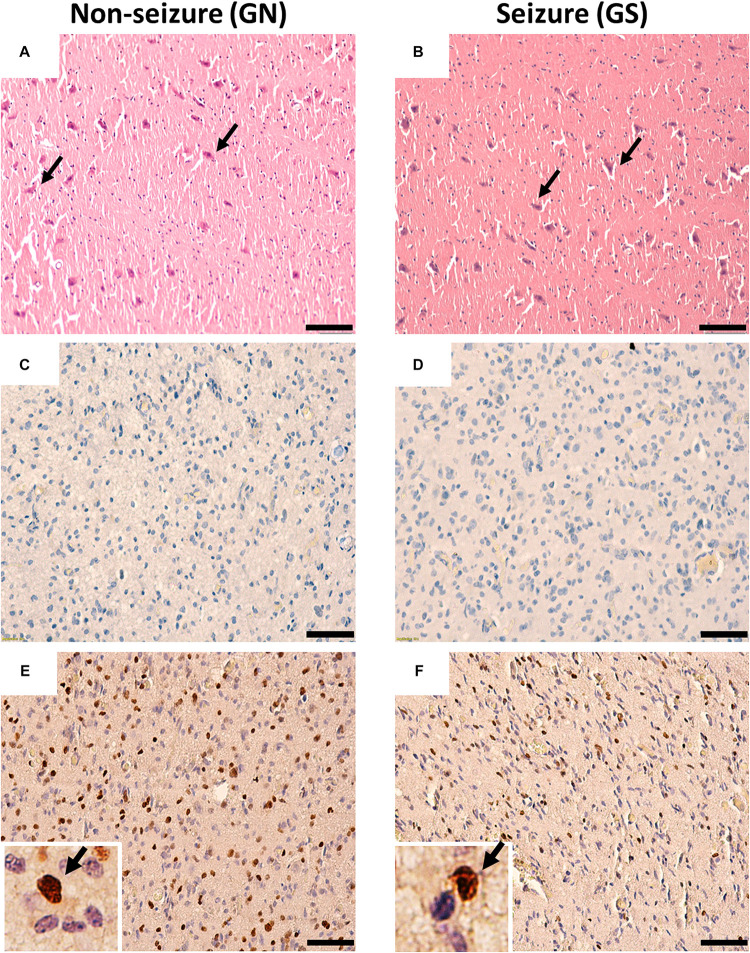
Cytoarchitecture of resected peritumoral specimens obtained from glioma patients without (GN) and without (GS) history of seizures. **(A,B)** H&E staining of peritumoral tissue sections from GN and GS patients showing mature pyramidal neurons (arrowheads), absence of gliosis, and tumor cell infiltrations. **(C,D)** Ki67 immuno-positive cells were absent in tissue sections from GN and GS patients. **(E,F)** Olig2 immuno-positive cells were scattered in tissue sections from GN and GS patients. OLig2 cells (brown) are marked with arrowheads (inset). The nuclei are stained with hematoxylin (blue). Scale bar, 50 μm.

The hematoxylin–eosin staining of the peritumoral tissue samples reveal scattered mature pyramidal neurons and the absence of glioma cell infiltration in both GS and GN groups (Figures 1A,B). Proliferating cells were absent in peritumoral cortex samples as evidenced by negligible Ki67 (Figures 1C,D) immuno-positive cells. Olig2 immuno-positive cells were scattered throughout in peritumoral cortex samples in both GS and GN groups (Figures 1E,F). However, no cytoarchitectural and immunohistochemical differences were observed between the two groups.

### Electrophysiological Parameters

**FIGURE 2:**
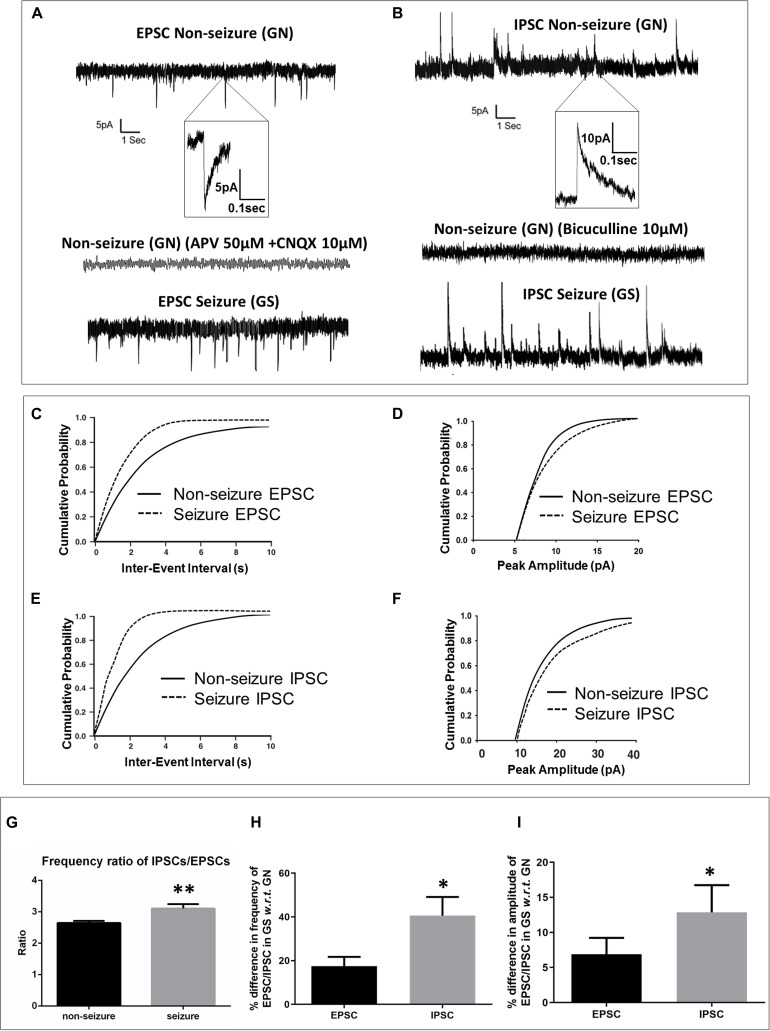
Spontaneous excitatory and inhibitory postsynaptic currents were enhanced in glioma patients with a history of seizures (GS). **(A)** Sample recordings of spontaneous excitatory postsynaptic currents (EPSCs) recorded from pyramidal neurons in the peritumoral sample obtained from glioma patients without history of seizures (GN). The inset shows a single EPSC event at an expanded time scale. The second trace shows the absence of any spontaneous EPSC following perfusion of the slice with glutamate receptor antagonists APV (50 μM) and CNQX (10 μM) for 10 min, proving that these events are mediated by glutamate receptors. The bottom trace shows sample recordings of spontaneous EPSCs recorded from pyramidal neurons in the peritumoral sample obtained from GS patients. The plots represent data from 15 neurons from 15 patients for GN (*n* = 15) and 11 neurons from 11 patients for GS (*n* = 11). **(C)** In GS patients, the cumulative distribution of inter-event interval displaced towards lower intervals (*p* = 0.004; Kolmogorov–Smirnov test, K–S test), while that of **(D)** peak amplitude displaced towards larger amplitudes (*p* = 0.026; K–S test). **(B)** Sample recordings of spontaneous inhibitory postsynaptic currents (IPSCs) recorded from pyramidal neurons in the peritumoral sample obtained from GN patients. The inset shows a single IPSC event at an expanded time scale. The second trace shows the absence of any spontaneous IPSC following perfusion of the slice with GABA_*A*_ receptor antagonist bicuculline (10 μM) for 15 min, proving that these events are mediated by GABA_*A*_ receptors. The bottom trace shows the sample recordings of spontaneous IPSCs recorded from pyramidal neurons in the peritumoral sample obtained from GS patients. **(E)** In GS patients, the cumulative distribution of inter-event interval displaced towards lower intervals (*p* = 0.0001; K–S test), while that of **(F)** peak amplitude displaced towards larger amplitudes (*p* = 0.009; K–S test). **(G)** The ratio of frequency of IPSCs/EPSCs was significantly high (*p* = 0.0031) in the peritumoral samples of GS patients as compared to that in GN patients. **(H)** The percentage difference in the frequency of spontaneous IPSCs in samples obtained from GS with respect to (w.r.t) that in the case of GN was significantly higher (*p* = 0.043) than that of spontaneous EPSCs in samples obtained from GS w.r.t that in the case of GN. **(I)** The percentage difference in the mean peak amplitude of spontaneous IPSCs in samples obtained from GS w.r.t that in the case of GN was also significantly higher (*p* = 0.028) than that of spontaneous EPSCs in samples obtained from GS w.r.t that in the case of GN. The data represented mean ± SEM. ^∗^*p* < 0.05; ^∗∗^*p* < 0.01; Mann–Whitney test.

The passive membrane properties of pyramidal neurons were similar in both groups (GN, *n* = 15; GS, *n* = 11; Table 2). sEPSCs were inward events and completely blocked following a 10-min bath perfusion of the slices with ACSF containing NMDA receptor antagonist APV (50 μM) and AMPA receptor antagonist CNQX (10 μM), implying that these events were mediated by glutamate receptors (Figure 2A). sEPSCs were unaffected by a bath perfusion with GABA_*A*_ receptor antagonist bicuculline (10 μM; Figure 2A), indicating that, under resting conditions, spontaneous glutamatergic activity was independent of GABAergic influence in the slice preparations. sIPSCs were outward currents and completely blocked following 15 min of bath perfusion of the slices with ACSF containing bicuculline (Figure 2B). The frequency and the amplitude of sEPSCs and sIPSCs were significantly increased in the peritumoral samples obtained from GS patients (GN, *n* = 15; GS, *n* = 11; Table 2). The cumulative distribution of inter-event intervals displaced toward a shorter interval, whereas that of peak amplitudes displaced toward a larger amplitude in the peritumoral samples obtained from GS patients (Figures 2C–F). The rise time and *τ*_*d*_ of sEPSCs and sIPSCs were not affected between the two groups (Table 2).

**TABLE 2:** Passive membrane properties and characteristics of spontaneous excitatory postsynaptic currents (sEPSCs) and spontaneous inhibitory postsynaptic currents (sIPSCs) recorded from pyramidal neurons in non-seizure and with seizure samples obtained from low-grade glioma patients.

Parameters	Non-seizure (*n* = 15)	Seizure (*n* = 11)	Statistics
**Passive membrane properties**			
Cell capacitance (pF)	158.8 ± 16	169.1 ± 13	*p* = 0.644
Input resistance (MΩ)	121.7 ± 18	110.5 ± 11	*p* = 0.469
**sEPSCs**			
Frequency (Hz)	0.67 ± 0.09	0.81 ± 0.08	*p* = 0.0058
Amplitude (pA)	12.23 ± 0.64	13.23 ± 0.78	*p* = 0.026
Rise time (ms)	2.1 ± 0.6	2.2 ± 0.9	*p* = 0.942
Decay time constant (τd, ms)	9.9 ± 1.3	10.1 ± 1.3	*p* = 0.916
**sIPSCs**			
Frequency (Hz)	1.790 ± 0.15	2.433 ± 0.33	*p* = 0.0001
Amplitude (pA)	21.96 ± 1.48	24.49 ± 2.59	*p* = 0.0053
Rise time (ms)	2.9 ± 0.8	2.7 ± 0.6	*p* = 0.853
Decay time constant (τd, ms)	34.7 ± 3.5	38.9 ± 4.7	*p* = 0.471

*Data are presented as mean ±SEM.*

We calculated the ratio of frequency of sIPSCs and sEPSCs. We found that the ratio of frequency was significantly increased in the peritumoral samples obtained from GS patients as compared to that in GN patients (2.65 ± 0.06 in GN patients *vs*. 3.11 ± 0.16 in GS patients; *p* = 0.0031; Figure 2G). We also calculated the percentage increase in the frequency as well as amplitude of sEPSCs and sIPSCs. We observed that the percentage increase in the frequency of sEPSCs was 17.49 ± 4.21% in the peritumoral samples obtained from GS with respect to (w.r.t) that in the case of GN, while the percentage change in the frequency of sIPSCs was 40.57 ± 8.53% in GS w.r.t. that in GN (*p* = 0.0431; Figure 2H). The mean peak amplitude of sEPSCs was 6.92 ± 2.30% higher in the peritumoral samples obtained from GS as compared to that in the case of GN. We also observed 12.88 ± 3.87% increase in the mean peak amplitude of spontaneous IPSCs in the peritumoral samples obtained from GS patients w.r.t. that of GN (*p* = 0.0280; Figure 2I).

We also compared the characteristics of spontaneous PSCs (sEPSCs/sIPSCs) between astrocytoma and ODG patients within GN (astrocytoma, *n* = 12; ODG, *n* = 3) and GS (astrocytoma, *n* = 4; ODG, *n* = 7) groups separately to investigate tumor-type-specific variation in synaptic transmission. However, no significant changes in spontaneous PSCs were observed between astrocytoma and ODG patients within the two groups (Supplementary Tables 1, 2).

## Discussion

Here we have demonstrated increased spontaneous glutamatergic and GABAergic synaptic activity onto pyramidal neurons in the peritumoral samples of GS patients as compared to that in GN patients. Under resting conditions, the frequency and the amplitude of sEPSCs recorded from pyramidal neurons in the peritumoral samples of GS patients were higher as compared to that in GN patients, which may be due to both presynaptic as well as postsynaptic glutamatergic activity. The levels of the extracellular glutamate around gliomas are not regulated and significantly higher than normal (Marcus et al., 2010). Due to the dysregulation of the extracellular glutamate level, the action potential-dependent presynaptic inputs were enhanced, leading to an increase in the frequency of sEPSCs. The glutamate receptors are also overexpressed in glioma cells (de Groot and Sontheimer, 2011). It might be possible that the higher level of the extracellular glutamate stimulates the postsynaptic glutamate receptors, which was reflected in the higher amplitude of sEPSCs in the peritumoral samples obtained from GS patients. We observed that the GS patients were positive for mutated IDH-1. The mutant IDH-1 reduces the conversion of α-ketoglutarate to D-2-hydroxyglutarate (D2HG), and the levels of D2HG are known to surpass 30 mM in IDH-1 mutant gliomas (Dang et al., 2009). D2HG, due to the structural resemblance, mimics the action of the glutamate and binds with the postsynaptic glutamate receptors which are upregulated and render those functionally hyperactive. Extracellular GABA levels are reported to be higher in the peritumoral tissues than in the tumor core (Bianchi et al., 2004), possibly due to the increased action potential-dependent GABA release as indicated by the higher frequency of sIPSCs in the peritumoral samples obtained from GS patients as compared to that in GN (Figures 2B,E). Furthermore, an increase in the amplitude of sIPSCs indicates a possible enhancement in the postsynaptic GABA_*A*_ receptor expression on the neurons in the peritumoral samples of GS patients (Figures 2B,F). It has been reported that, in the peritumoral samples obtained from patients with glioma having seizures, GABA-mediated inhibition remains impaired, and GABA acts as a depolarizing neurotransmitter (Köhling and Avoli, 2006; Smits et al., 2012; Chang et al., 2013). The GABA receptor activity depends on the neuronal chloride homeostasis. Under physiological conditions, the intracellular chloride concentration remains low due to the activation of KCC2 which transports chlorides out of the neurons and the suppression of NKCC1 which transports chloride ions inside the neurons (Ben-Ari et al., 2007). The altered expression of KCC2/NKCC1 is associated with a disturbance in chloride homeostasis. The reduced expression of KCC2 and the enhanced activity of NKCC1 contributes to the altered neuronal chloride homeostasis in glioma with seizure (Ernest et al., 2005; Haas and Sontheimer, 2010; Garzon-Muvdi et al., 2012). The intracellular chloride concentration in tumor cells is generally 10-fold higher than normal due to the overexpression and activity of NKCC1 (Huberfeld and Vecht, 2016). The increased NKCC1 activity, which drives neuronal chloride influx causing the depolarizing effect of GABA, may not be counter-balanced by KCC2 due to its lower expression in glioma tissues (Conti et al., 2011; Pallud et al., 2014; Huberfeld and Vecht, 2016). Furthermore, NMDA receptor activity is known to inhibit KCC2 expression (Lee et al., 2011), and the hyperactive glutamate receptor activity, as observed in our study, could trigger the dysregulation of chloride homeostasis in patients with glioma. Consequently, opening of GABA receptors results in efflux of chloride ions from the pyramidal neurons, leading to paradoxical depolarization and GABA_*A*_ receptor-mediated interictal epileptogenicity in patients with low-grade glioma (Conti et al., 2011; Pallud et al., 2014). Further studies are needed to understand the role of the ratio of NKCC1/KCC2 to epileptogenicity in patients with low-grade gliomas. The higher ratio of frequency of GABAergic/glutamatergic activities in the peritumoral samples of GS patients indicated reinforced GABA_*A*_ receptor activity under basal conditions. Our study suggests that, in addition to the hyperactive glutamatergic excitatory drive, the dysregulated chloride homeostasis and the paradoxical excitatory behavior of GABA_*A*_ receptors cause an imbalance between excitation and inhibition, which may contribute to the spontaneous epileptiform discharges and be possibly responsible for the recurrent seizures in GS patients. However, we could not observe a tumor-specific variation in GABAergic/glutamatergic activities in the peritumoral samples. We speculate that, despite the small sample size, the hyperexcitable synaptic transmission observed in GS patients was not due to tumor specificity but rather due to the inherent cellular and molecular alterations of the peritumoral cortex. To our knowledge, this is the first report comparing synaptic transmission in peritumoral samples obtained from low-grade glioma patients with a history of seizures at presentation with those of patients without any history of seizures. Further studies correlating altered synaptic transmission with age, gender, and tumor location in patients with low-grade gliomas in a large cohort will be needed to confirm these findings.

This human study has a few limitations. All GS patients were on a combination of AEDs that may have affected both glutamatergic and GABAergic activities recorded from pyramidal neurons. AEDs act through the suppression of voltage-gated sodium or calcium channels, potentiation of GABA, or inhibition of glutamate receptors (Miziak et al., 2020). In *in vitro* mouse hippocampal slices, AEDs like carbamazepine, phenytoin, and valproate suppressed epileptiform discharges (paroxysmal depolarization shifts), but levetiracetam did not show any effect (Lin et al., 2017). *In vivo* studies have also reported the modulation of neuronal activity due to AEDs. Data indicate that diazepam (Pitkänen et al., 1996) and valproate (Bolanos et al., 1998; Brandt et al., 2006) exhibited moderate to strong neuroprotective effects in rat kindling and chemical-induced epilepsy models. However, carbamazepine was devoid of any neuroprotective effect (Halonen et al., 1996). Levetiracetam alone and in combination with other AEDs proved effective to prevent epileptogenesis following status epilepticus (Sugaya et al., 2010; Schidlitzki et al., 2020). Moreover, inherent heterogeneity and a different tumor behavior that differs between ODGs and astrocytoma may also contribute to the difference in synaptic activity between GS and GN groups and need to be studied in a larger cohort. The infiltration of tumor cells in the peritumoral cortex cannot be ruled out, which may vary between the GS and GN groups. The possibility of the influence of neurodevelopmental factors on synaptic transmission cannot be ruled out as a pediatric population was involved in this study.

## Conclusion

This study suggests a tight association between altered glutamatergic and GABAergic activity and spontaneous epileptiform discharges in the peritumoral samples of patients with low-grade glioma presenting with seizures.

## Data Availability Statement

The original contributions presented in the study are included in the article/Supplementary Material, further inquiries can be directed to the corresponding author.

## Ethics Statement

The studies involving human participants were reviewed and approved by Institutional Ethics Committee, All India Institute of Medical Sciences (AIIMS), New Delhi. Written informed consent to participate in this study was provided by the participants’ legal guardian/next of kin.

## Author Contributions

SD and JB designed and performed the research and analysed the data. SD, RD, and JB wrote the manuscript. RD and PC designed the research and provided the clinical samples. AB and MT designed the research. MS analysed the histopathological features and IHC. All authors read and approved the final manuscript.

## Conflict of Interest

The authors declare that the research was conducted in the absence of any commercial or financial relationships that could be construed as a potential conflict of interest.
