# Supplementary material for: Altered Spontaneous Glutamatergic and GABAergic Activity in the Peritumoral Cortex of Low-Grade Gliomas Presenting With History of Seizures
Source: Front Neurosci. 2021 Jun 28;15:689769. doi: 10.3389/fnins.2021.689769 (PMC8273299; doi:10.3389/fnins.2021.689769)
Supplement: Supplementary file 1 [file Table_1.DOCX]

**Table S1: Characteristics of EPSCs and IPSCs recorded from pyramidal neurons in samples obtained from low-grade** **astrocytoma and oligodendroglioma patients without seizure. Data are presented as a mean ± SEM.**

| Parameters | Astrocytoma (n=12) | Oligodendroglioma (n=3) |
| --- | --- | --- |
| Spontaneous EPSCs | | |
| Frequency (Hz) | 0.67 ± 0.04 | 0.70 ± 0.05 |
| Amplitude (pA) | 12.03 ± 0.59 | 12.92 ± 0.54 |
| Rise time (ms) | 2.0 ± 0.4 | 2.2 ± 0.7 |
| Decay time constant (τ_d_, ms) | 10.2 ± 0.9 | 9.3 ± 1.6 |
| Spontaneous IPSCs | | |
| Frequency (Hz) | 1.75 ± 0.1 | 1.93 ± 0.26 |
| Amplitude (pA) | 21.78 ± 1.53 | 22.63 ± 1.27 |
| Rise time (ms) | 2.9 ± 0.6 | 2.8 ± 0.9 |
| Decay time constant (τ_d_, ms) | 33.5 ± 3.1 | 34.8 ± 5.1 |
