# Supplementary material for: Altered Spontaneous Glutamatergic and GABAergic Activity in the Peritumoral Cortex of Low-Grade Gliomas Presenting With History of Seizures
Source: Front Neurosci. 2021 Jun 28;15:689769. doi: 10.3389/fnins.2021.689769 (PMC8273299; doi:10.3389/fnins.2021.689769)
Supplement: Supplementary file 2 [file Table_2.DOCX]

**Table S2:** **Characteristics of EPSCs and IPSCs recorded from pyramidal neurons in samples obtained from low-grade astrocytoma and oligodendroglioma patients with seizure. Data are presented as a mean ± SEM.**

| Parameters | Astrocytoma (n=4) | Oligodendroglioma (n=7) |
| --- | --- | --- |
| Spontaneous EPSCs | | |
| Frequency (Hz) | 0.78 ± 0.06 | 0.79 ± 0.05 |
| Amplitude (pA) | 13.03 ± 0.90 | 13.33 ± 0.76 |
| Rise time (ms) | 2.3 ± 0.5 | 2.0 ± 0.8 |
| Decay time constant (τ_d_, ms) | 10.1 ± 1.7 | 10.1 ± 1.4 |
| Spontaneous IPSCs | | |
| Frequency (Hz) | 2.24 ± 0.37 | 2.56 ± 0.27 |
| Amplitude (pA) | 23.53 ± 2.89 | 25.04 ± 2.47 |
| Rise time (ms) | 2.8 ± 0.5 | 2.6 ± 0.3 |
| Decay time constant (τ_d_, ms) | 38.7 ± 5.1 | 39.1 ± 4.9 |
